# The diagnosis of multiple sclerosis and the clinical subtypes

**DOI:** 10.4103/0972-2327.58276

**Published:** 2009

**Authors:** Barrie J. Hurwitz

**Affiliations:** Departments of Medicine, Neurology, and Community and Family Practice, Duke University Medical Center, Box 3184 DUMC, Durham, NC 27710, USA

**Keywords:** Multiple Sclerosis, Clinical subtypes, diagnosis, diagnostic criteria

## Abstract

The diagnosis of multiple sclerosis (MS) requires objective findings referable to the central nervous system. A wide differential diagnosis often has to be considered. Magnetic resonance imaging and electrophysiologic and cerebrospinal fluid studies can all contribute to an early definitive diagnosis. The McDonald diagnostic criteria for MS (2005) are the currently recognized MS diagnostic criteria. The clinical subtypes of MS and their diagnosis are discussed in this article. Being informed of the diagnosis may be a stressful experience for the patient and this is also dealt with.

## Introduction

Multiple sclerosis (MS) is a disease of the central nervous system (CNS), which affects the brain, spinal cord, and the optic nerves. MS does not directly affect the peripheral nerves. The usual age of onset is between 15 and 50, although cases have been reported to occur from the first to the eighth decades.[1] It is the commonest nontraumatic cause of neurologic disability in young adults.[2] The prevalence of MS appears to be increasing and is significantly greater in women than in men. Inflammation, demyelination, and axonal damage characterize MS lesions. The clinical onset is usually subacute, evolving over hours to days. The symptoms and signs depend upon the location of the MS lesions in the CNS. Some of the symptoms of MS may be very nonspecific, while others may be very suggestive of the diagnosis, e.g., bilateral internuculear ophthalmoplegia occurring in isolation. Thus, the diagnosis of MS may be straightforward and easy in some patients yet elusive and challenging in others.[3] Modern diagnostic investigations [MRI imaging, evoked potential electrophysiologic studies, and cerebrospinal fluid (CSF) immunologic studies] have all contributed toward accurate and early diagnosis of MS in many patients.[4]

## The diagnosis of MS

The majority of MS patients (approximately 85%) present with subacute relapses or attacks, with symptoms and signs referable to the CNS.[5] The relapse/attack is followed by a complete or partial remission/return to normal, only to be followed at a future date by another relapse usually in a different CNS location, thus presenting as relapsing and remitting MS (RRMS). The first such attack is referred to as a clinically isolated syndrome (CIS). Some patients (approximately 15%) present with a gradually progressive course, without an initial well-defined attack. This is termed primary progressive MS (PPMS). Most of these patients present with features of a spinal cord syndrome.

Modern diagnostic criteria for MS require the following:

Objective evidence of two separate CNS lesions compatible with MS and separated both in space and time of occurrence.

Other potential causes for the CNS lesions must have been ruled out or excluded.

These principles were first established with the Schumacher criteria in 1965, when the diagnosis of MS was entirely clinical.[6] In 1983, the Poser criteria replaced the Schumacher criteria for the diagnosis of MS. The Poser criteria incorporated paraclinical evidence that was now available from diagnostic studies developed during the 1970s. These criteria were based on a detailed literature review at that time and represented the views of 25 MS experts.[7] The Poser criteria were developed to guide clinical trials but were subsequently also used in the clinical setting. ‘Laboratory-supported definite multiple sclerosis’ was a new term introduced by the Poser criteria. In 2000, an international panel was convened to develop new diagnostic criteria for MS and the panel's recommendations became known as the McDonald criteria[8] The McDonald diagnostic criteria (2001 and 2005) allow an earlier and often more accurate diagnosis of MS to be made by utilizing MRI, evoked potentials, and CSF immunologic changes to fulfill criteria for dissemination in space and time; these criteria are shown in detail in Tables 1, 2 and 3.[8,9] The McDonald diagnostic criteria for PPMS also allow for an earlier diagnosis to be made, as compared to the older criteria, and are shown in Table 1.

**Table 1:** The Diagnosis of MS: McDonald criteria - 2005

Clinical presentation	Additional data needed for MS diagnosis
Two or more attacks; objective clinical evidence of two or more lesions	None
Two or more attacks; objective clinical evidence of 1 lesion	Dissemination in space, demonstrated by: MRI [Table 2] or Two or more MRI-detected lesions consistent with MS plus positive CSF or Further clinical attack at a different site later
One attack; objective clinical evidence for two or more lesions	Dissemination in time, demonstrated by: MRI [Table 3] or Second clinical attack
One attack; objective clinical evidence of one lesion (monosymptomatic presentation; CIS)	Dissemination in space, demonstrated by: MRI [Table 2] or Two or more MRI-detected lesions consistent with MS plus positive CSF and Dissemination in time, demonstrated by: MRI [Table 3] or Second clinical attack
Insidious neurologic progression suggestive of MS (PPMS)	1-year disease progression (retrospectively or prospectively objectively determined) two of the following: Positive brain MRI (nine T2 lesions or four or more T2 lesions with positive VEP) Positive spinal cord MRI (two focal T2 lesions) Positive CSF

**Table 2:** MRI findings demonstrating dissemination in space - McDonald criteria - 2005

Any three of the following: ≥1 Gadolinium-enhancing lesion of the brain or spinal cord OR nine T2 hyperintense lesions on the brain or spinal cord if there are no gadolinium-enhancing lesions ≥1 Infratentorial or spinal cord lesion ≥1 Juxtacortical lesion ≥3 Periventricular lesions

**Table 3:** MRI Findings to demonstrate dissemination in time — McDonald criteria -2005

Timing	Type of Lesion	Site
≥3 Months after initial episode onset Or-	New gadolinium-enhancing T 1 lesion	Separate from site of initial event
Any time after a reference (baseline) scan done ≥30 days after initial episode onset	New T2 lesion	Separate site

## The differential diagnosis

The differential diagnosis includes many conditions [Table 5] and will vary according to the clinical presentation. The differential diagnosis will dictate which investigations may be needed.[10] A detailed history and general medical examination as well as a complete neurologic examination is essential since an initial clinical finding on the neurologic examination referable to the CNS remains a prerequisite for the diagnosis.[8,9] The history may reveal previous symptoms suggestive of MS, such as unilateral visual loss, blurred or double vision, Lhermitte sign, or a motor or sensory disturbance in a CNS distribution. These symptoms are helpful in suggesting a diagnosis of MS but cannot be part of the diagnostic criteria unless there is documented evidence of objective deficits at these times.[8,9]

**Table 4:** 2006 Swanton criteria

MRI findings demonstrating dissemination in space
At least one lesion in at least two of the following regions: Periventricular Juxtaortical Infratentiorial Spinal cord
Note: In cases with brain stem and spinal cord syndromes, all lesions within the symptomatic regions are excluded and cannot be used to fulfill these criteria.
MRI findings demonstrating dissemination in time
One or more new T2 lesion on a 3 month follow-up scan

**Table 5:** The differential diagnosis of MS

Inflammatory/autoimmune	Demyelinating
Systemic lupus erythematosis	Neuromyelitis optica (Devic)
Sarcoidosis	Idiopathic transverse myelitis
Sjogren syndrome	Acute disseminated encephalomyelitis
Wegener granulomatosis	Optic neuritis in isolation
Susac syndrome	Schilder disease
Behcet syndrome	CNS neoplasms
Infectious	CNS lymphoma
Progressive multifocal leukoencephalopathy	Nutritional disorders
HIV-related disorders	Vitamin B12 deficiency
HTLV1	Cerebrovascular disease
Genetic/hereditary	Hypertensive
Adrenomyeloneuropathy	Vasculitis
CADASIL	Atherosclerotic
Hereditary spastic paraparesis	Migraine

Once the clinical examination has revealed evidence of an MS attack, the usual next step is an MRI brain scan. If the patient presents with a spinal cord syndrome suggestive of MS, then MRI of both brain and spinal cord should be done. The location, size, and orientation of the MRI lesions may often suggest MS but, in the absence of a suggestive clinical presentation, these MRI lesions are by themselves not very specific for MS.[11] If necessary visual evoked potentials (VEP) may also reveal a clinically unaffected site helping fulfill dissemination in space criteria. CSF examination is often very suggestive of MS, showing an increase in IgG synthesis rate and/or index and the presence of oligoclonal bands of IgG which are not seen in the serum. However, a differential diagnosis still needs to be considered because conditions such as viral encephalitis, syphilis, subacute sclerosing panencephalitis, and acute disseminated encephalomyelitis may also produce these CSF abnormalities. These paraclinical findings therefore, whilst being very suggestive of an MS diagnosis, are not always specific for it, and it is pertinent to reiterate that the diagnosis of MS remains primarily a clinical one and a differential diagnosis must always be considered.[6–9]

## Clinically isolated syndrome (CIS)

The McDonald diagnostic criteria still require that the first MS attack – known as a CIS – be clinical, with features typical or suggestive of MS and with objective abnormalities (lasting at least 24 h) on neurologic examination. Typical presentations include optic neuritis, usually unilateral and painful; a partial brainstem syndrome, which may include isolated cranial nerve deficits (including trigeminal neuralgia) but more typically internuclear ophthalmoplegia (unilateral or bilateral); partial cerebellar syndromes; and partial motor or sensory deficits. Incomplete and/or asymmetric transverse myelitis is another, not uncommon, presentation. If the CIS presentation is truly monofocal as judged on the clinical examination then, according to the McDonald criteria, dissemination in space should be present for a diagnosis of CIS to be made. This condition can be fulfilled by finding an abnormal VEP (if the clinical presentation is not visual) and MRI lesions [Table 2]. An isolated clinical presentation such as optic neuritis, with normal MRI scan of the brain (with and without contrast) has a very low risk of converging to MS, being up to 100% at 5 years and 22% at 10–14 years.[12–14] As there are many conditions that may mimic MS, a differential diagnosis needs to be considered in all patients presenting with CIS. Conditions that may mimic MS are listed in Table 5. Depending upon the nature of the CIS, all or some of these illnesses need to be considered and excluded before a diagnosis of CIS or a first MS attack can be made.

The importance of early recognition of CIS is borne out by the results of therapeutic trials in this condition, which show that immunomodulatory therapy introduced early is superior to placebo in terms of prolonging the time to next attack, reducing the amount of new disease seen on MRI scans, and in slowing disability progression as well as cognitive impairment.[15–17] Nonspecific complaints of fatigue; fluctuating sensory symptoms, without objective evidence of abnormality; depression; and mild cognitive impairment are not sufficient by themselves to fulfill criteria for a CIS indicative of first MS attack. Recently, reports of such patients with abnormal MRI scans have been published[18,19] and these cases have been termed ‘radiologically isolated syndromes.’[19] Follow-up of these small (30 patients in each) cohorts over a number of years has revealed that up to 37% develop a CIS or MS at 5 years. Thus, whilst a diagnosis of CIS indicative of a first MS attack cannot be made in such patients, they should be followed closely in the clinic with repeated examinations, both clinical and with MRI, to ensure that clinical and radiological progression does not occur.

## Relapsing and remitting multiple sclerosis (RRMS)

The 2005 revisions to the McDonald criteria for the diagnosis of RRMS are shown in Tables 1, 2, and 3. The criteria clearly indicate the need for objective evidence, both clinical and radiological or with evoked potentials, for proving dissemination in both space and time to be present when a diagnosis of RRMS is made. These MRI criteria, based on the Barkoff MRI criteria, have revealed an accuracy of 79% for dissemination in space and 82% for dissemination in time.[20] Utilization of the McDonald criteria rather than the Poser criteria allows for an earlier diagnosis of RRMS to be made.

Swanton *et al*.[21] in 2005 recommended simplified MRI criteria for proving dissemination in space and time to diagnose RRMS [Table 4]. These Swanton MRI criteria necessitate that the ‘CIS should be unambiguously typical of those seen in MS, e.g., unilateral optic neuritis, bilateral internuclear ophthalmoplegia, or partial myelopathy.’[21] These criteria were recently compared to the McDonald MRI criteria for dissemination in space and time in a retrospective cohort of 282 patients who had converted from CIS to clinically definite MS (two clinical relapses) and both criteria were found to have equal accuracy.[20] Polman, in reviewing the early diagnosis of MS, noted that the most specific finding for MS in this analysis was a new T2 lesion occurring more than 30 days after a baseline scan (McDonald criteria - 2005); he reiterates that objective evidence of dissemination in time remains crucial for a diagnosis of MS to be made.[22] Whilst RRMS may present with two clinical attacks referable to the CNS, separate in both time and space, and with normal MRI, VEP, and CSF, this is not common, and extreme caution should be used and a wide differential considered in such a situation.[8,9]

RRMS is characterized by recurrent attacks or relapses, which vary in frequency and severity, but there is a stable baseline between relapses. A relapse may however never completely revert to normal and many patients are left with a residual disability.

## Benign multiple sclerosis

Benign MS is usually a subset of RRMS and comprises patients who accumulate little disability over many years and remain very functional. Autopsy evidence has indicated undiagnosed MS in some patients without clinical features of MS, thereby indicating a benign course. There are no reliable predictors of benign MS, although some have suggested that it is more commonly seen in women, with a younger age of onset and with initial presentations of optic neuritis or sensory dysfunction[23] Studies reveal that of patients with diagnosed MS and classified as benign after 10 years, with an EDSS of 3 or less, only 55% or less remain benign over the subsequent 10 years.[23,24] Benign MS should, therefore, probably only be diagnosed in retrospect.

## Secondary progressive multiple sclerosis (SPMS)

SPMS is characterized by at least one relapse followed by progressive clinical worsening over time. This progressive course may develop slowly after an initial CIS, but more commonly follows a period of well-defined RRMS. It therefore follows that a well-defined CIS is the minimum prerequisite for this type of MS to be considered. The course is usually steadily progressive, but in some patients there may be periods of relative stability. The progressive course may also be punctuated by intermittent relapses. SPMS is most typically seen 3 or more years after the onset of RRMS, with 90% of RRMS patients becoming SPMS after 25 years.[5] Patients usually become more disabled as SPMS progresses, and this form of MS accounts for most of the disability seen with this illness.

## Primary progressive multiple sclerosis (PPMS)

This is an uncommon form of MS, accounting for about 15% of the MS population.[5] There is a slowly progressive disability from the onset and clinically most patients present with features of a progressive myelopathy or with progressive cerebellar dysfunction. A definitive diagnosis may be difficult in many of these patients with this type of presentation, but strict utilization of the McDonald criteria should help avoid diagnostic errors. According to the criteria, for the diagnosis of MS there must be a minimum of 1 year's disease progression with at least two of the following: a positive brain MRI, a positive spinal cord MRI, and positive CSF findings.[25] Some patients seem to plateau after some time; most, however, progress relentlessly. PPMS are more commonly seen in males and in older patients.

An uncommon subtype, progressive relapsing MS (PRMS), can also occur, where the gradual progressive course may, over time, be punctuated by one or more relapses.

## Communicating the diagnosis

Receiving a diagnosis of MS, a potentially disabling illness, may be very difficult for the patient. The diagnosis should be communicated by the diagnosing physician, usually a neurologist, with empathy; the physician must use clear, everyday language to explain the illness and must give the patient enough time to discuss its implications and possible prognosis. Most patients prefer to have a relative or close friend with them at this time.[26] The physician should have an adequate knowledge of MS. An explanation of the patient's symptoms should be given, as well as information about disease-modifying drugs and current symptom management. Written information about MS and/or referral to a local MS society or Internet site such as www.nationalmssociety.org, would be helpful, as well as an early follow-up appointment with the diagnosing neurologist. A recent (2003) survey of 434 patients and 80 neurologists found that only half of the patients believed that their neurologist helped them understand the illness and informed them of any form of therapy at the time of the initial diagnosis.[27] Effective communication by the neurologist, especially initially, may have a very positive effect on how the patient accepts and understands the diagnosis and copes with the illness in the future. A knowledgeable nurse or social worker can provide additional help and resources at such a critical time for the patient. A detailed outline of how to provide such support initially and upon follow-up has been provided by Vitali.[28]
